# *De novo* sequencing and comparative analysis of leaf transcriptomes of diverse condensed tannin-containing lines of underutilized *Psophocarpus tetragonolobus* (L.) DC

**DOI:** 10.1038/srep44733

**Published:** 2017-03-21

**Authors:** Vinayak Singh, Ridhi Goel, Veena Pande, Mehar Hasan Asif, Chandra Sekhar Mohanty

**Affiliations:** 1 Plant Molecular Biology and Genetic Engineering Division, CSIR-National Botanical Research Institute, Lucknow-226 001 Uttar Pradesh, India; 2 Department of Biotechnology, Kumaun University, Nainital, Uttarakhand, 263001 India

## Abstract

Condensed tannin (CT) or proanthocyanidin (PA) is a unique group of phenolic metabolite with high molecular weight with specific structure. It is reported that, the presence of high-CT in the legumes adversely affect the nutrients in the plant and impairs the digestibility upon consumption by animals. Winged bean (*Psophocarpus tetragonolobus* (L.) DC.) is one of the promising underutilized legume with high protein and oil-content. One of the reasons for its underutilization is due to the presence of CT. Transcriptome sequencing of leaves of two diverse CT-containing lines of *P. tetragonolobus* was carried out on Illumina Nextseq 500 sequencer to identify the underlying genes and contigs responsible for CT-biosynthesis. RNA-Seq data generated 102586 and 88433 contigs for high (HCTW) and low CT (LCTW) lines of *P. tetragonolobus*, respectively. Based on the similarity searches against gene ontology (GO) and Kyoto encyclopedia of genes and genomes (KEGG) database revealed 5210 contigs involved in 229 different pathways. A total of 1235 contigs were detected to differentially express between HCTW and LCTW lines. This study along with its findings will be helpful in providing information for functional and comparative genomic analysis of condensed tannin biosynthesis in this plant in specific and legumes in general.

There are several underutilized and neglected legumes with great potentiality in terms of its nutritional values. One among them is *Psophocarpus tetragonolobus* (L.) DC., the “soybean of tropics^1^”. This plant has exceptionally high protein content and has been suggested as a potential food source for the tropics^2^. Almost all parts of this plant like: leaves, pods, seeds and tubers are edible and rich in protein. Winged bean (*Psophocarpus tetragonolobus* (L.) DC. is synonymously known as: goa bean or princess bean. It is a diploid (2n = 2x = 18), self-fertilizing legume with diverse use^3^. Winged bean is a tropical legume and is listed under unutilized or under-exploited category^4^.

Apart from its nutritional value, *P. tetragonolobus* contains some pharmacologically active anti-nutrients like: inhibitors, haemagglutinins along with condensed tannins (proanthocyanidins). Condensed tannin (CT) is reported to be present in the seeds of *P. tetragonolobus*^5^,^6^,^7^,^8^. Modifications such as hydroxylation, methylation, acylation and glycosylation result in various kinds of flavonoid colours^9^,^10^,^11^,^12^,^13^. CTs are synthesized via phenylpropanoid and the flavonoid pathways and are widespread in the plant kingdom. It confers protection against predation and pathogen attack^14^,^15^. They contribute to the bitter flavor and astringency and have a significant influence on mouth feel^16^. But, till date, there are very few reports on condensed tannins (CTs) and its biosynthesis in any of the underutilized plant. The herbages reported to contain CT include sainfonin (*Onobrychis viciifolia*), horse foot trefoil (*Trifolium arvense* L.), birds foot trefoil (*Lotus corniculatus*) and grasslands maku lotus (*Lotus pedunculatus*). The presence of CT in over 8–10% of dietary dry matter, markedly reduces the nutritive value of the browse shrub mulga (*Acacia aneura*)^17^. However, the present study focuses on the role of various genes or contigs in the biosynthesis of CT in this underutilized *P. tetragonolobus.* The present study and its finding will open up new avenue for understanding the molecular basis of biosynthesis of CT for further improvement of this crop.

In recent years, several studies have successfully reported the generation of transcriptome data for gene discovery in non-model plants for which no reference genome sequences are available^18^. Due to the availability of quick, low cost sequencing and high quality annotation and assembly tools, it has become possible to analyze and understand the genome of non-model plants. As a non-model legume *P. tetragonolobus* is carried out for transcriptome sequencing. Condensed tannin is considered as an anti-nutrient. Effective and optimum reduction of CT concentration from the plants will promote the lesser-known underutilized legumes for further consumption where there is an ever increasing demand for food and nutrition security. So, in order to genetically-improve this plant, it is needed to identify and understand the metabolic pathways and the underlying factors or genes regulating the biosynthesis of CT.

Most tracheophytes synthesize condensed tannins in the vacuole as uniformly stained deposits and are termed tannin accretions, these appear as the lining in the inner face of the tonoplast and are formed in the thylakoid-derived organelles in the name “tannosomes”. These are packed in membrane-bound shuttles, it has been suggested that these shuttles agglomerate into tannin accretions. Further, the tannosomes travel from the plastid towards the vacuole in multiple membrane-bound shuttles. So, leaf is the centre of condensed tannin biosynthesis in plants. So, the leaf-tissues of *P. tetragonolobus* are targeted for transcriptome sequencing.

Recently, *de novo* assembled trancriptomes of four underutilized legumes namely: *Lablab purpurea* (Malwi origin), *Lathyrus sativus* (Indian origin), *Psophocrapus tetragonolobus* (Nigeria origin) and *Vigna subterranean* (Africa origin) were carried out and 1139 SSRs out of 33,042 genes and 49,280 contigs of *P. tetragonolobus* were identified^19^. Recently the report identifying SSRs and SNPs among the *de novo* assembled transcriptome data of two Sri Lankan accessions of *P. tetragonolobus* had been published^20^. However, most of their studies related to the detection of SSR and SNP molecular markers from transcriptome data of *P. teragonolobus* from Nigerian and Sri Lankan origin. It was carried out on Roche 454 sequencing platform. But, the present study focuses on the biochemical screening of *P. tetragonolobus* for condensed tannin and mining contigs or genes responsible for biosynthesis of condensed tannin in *P. tetragonolobus*. This is the first report of transcriptome study of phenylpropanoid biosynthesis pathway responsible for condensed tannin biosynthesis in *P. tetragonolobus*. The study may pave path for further manipulation of genes or transcription factors for reducing the content of condensed tannin in the plant to make it more acceptable.

## Result

### Quantitative and qualitative estimation of condensed tannin in *P. teragonolobus*

**Figure 1:**
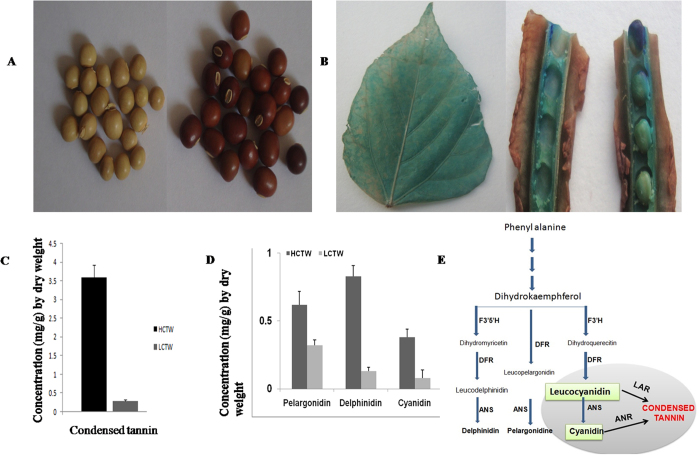
Vanillin Assay and HPLC analysis of HCTW and LCTW plant. (**A**) low condensed tannin winged bean (LCTW) and high condensed tannin winged bean (HCTW) seeds (**B**) Localization of condensed tannin in leaf and pod contains seed of HCTW plant (**C**) Estimation of condensed tannin through vanillin assay (**D**) Estimation of major metabolites of condensed tannin through HPLC of both HCTW and LCTW plant. (**E**) Flavonoid pathway showing the major compound and gene responsible for condensed tannin biosynthesis.

Quantitative estimation of CT through vanillin assay was carried out among 100 accessions of *P. tetragonolobus* collected from different parts of the world (Supplementary Table 1). The range of CT varies between (3.59 ± 0.31)mg/g DW and (0.27 ± 0.04) mg/g DW (Fig. 1C) and accordingly denoted as high condensed tannin-containing winged bean (HCTW) for accession (EC-38956-1 from Papua New Guinea) and low condensed tannin-containing winged bean (LCTW) for accession (EC-178268 from Malaysia). Histochemical staining of the plant-parts with dimethylamminocinnamaldehyde (DMACA) provided a clear picture of its deposition in different tissues as it developed a deep-blue colour when stained with DMACA (Fig. 1B). Flavan-3-ol (catechin) is inferred to be the basic monomeric unit as per the analysis of the plant-tissues on HPLC-platform and reported the presence of pelargonidin (0.62 ± 0.1 mg/g DW), delphinidin (0.83 ± 0.08 mg/g DW) and cyanidin (0.38 ± 0.06 mg/g DW) in HCTW and pelargonidin (0.32 ± 0.04 mg/g DW), delphinidin (0.13 ± 0.03 mg/g DW), cyanidin (0.08 ± 0.04 mg/g DW) in LCTW *P. tetragonolobus* (Fig. 1D).

### Transcriptome sequencing, *de novo* assembly and functional annotation of contigs

**Figure 2:**
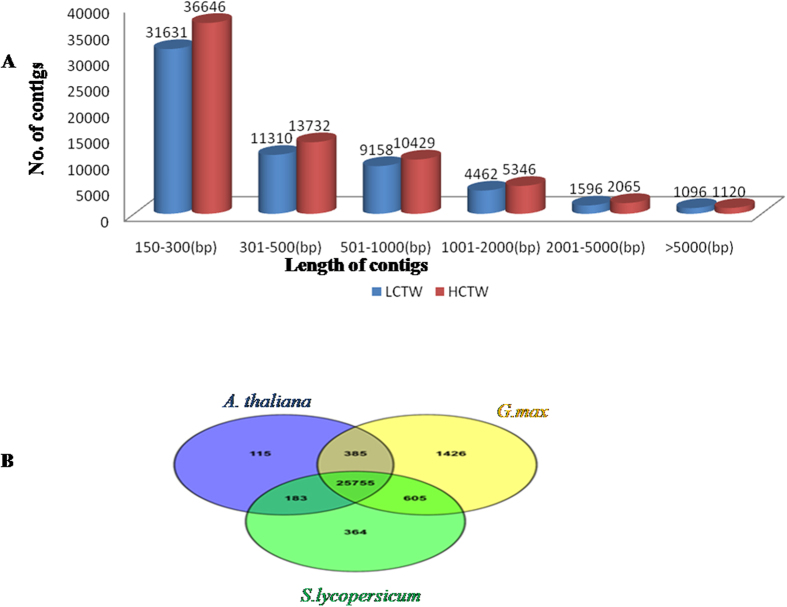
Secondary assembled Transcript length distribution. (**A**) Count of Contigs vs Lengh (bp) of Contig (**B**) venn diagram representing number of transcript data sets unique and shared data set from *G. max, S. lycopersicum* and *A. thaliana* after assembling all data of both LCTW and HCTW.

The complementary DNA (cDNA) libraries prepared from the diverse CT leaf tissues of winged bean were sequenced using Illumina Nextseq500 platform. Paired-end sequencing-by-synthesis (SBS) yielded raw data 13901780 (13.9 million) for VS1 and 30155788 (30.15 million) for VS2 in HCTW line and 9090662 (9.09 million) for VS3 and 10394022 (10.3 million) reads for VS4 in LCTW line of *P. tetragonolobus*. VS1 and VS2 are the replicated leaf transcriptome data for high condensed tannin-containing (HCTW) line of winged bean and VS3 and VS4 represented the duplicate low condensed tannin (LCTW) lines of winged bean. After trimming, number of high quality reads in HCTW line were (VS1) 12604265 (84.01%) and (VS2) 27769919 (92.09%) and LCTW line were (VS3) 8098380 (89.08%) and (VS4) 9518980 (91.58%). The replicated data of HCTW (VS1 and VS2) and LCTW (VS3 and VS4) were clustered and *de novo* assembled together using Velvet assembler with a hash length of 45. This generated 102586 and 88433 contigs for HCTW and LCTW line of winged bean, respectively. Contig generation was carried out using Oases-0.2.08 with same hash length that resulted in 87925 and 69464 contigs for HCTW and LCTW lines of *P. tetragonolobus*, respectively. In both cases, average contig lengths were of 713.1 bp and 736.0 bp with N50 values of 1466 and 1567 for HCTW and LCTW lines, respectively (Table 1). Distribution of assembled contig length ranged from 151 to >87925 bases. Maximum number of contigs was of (150–300) bp size with 36646 contigs followed by 13732 contigs of (301–500) bp size in HCTW and LCTW lines of *P. tetragonolobus*. About (150–300) bp size contigs were highest in number (31631 contigs) followed by 11310 contigs of (301–500) bp size. In both cases, some of the contigs, 1120 in HCTW and 1096 in LCTW were more than 5000 bp size (Fig. 2A). The contigs from both *P. tetragonolobus* samples were clustered using CD-HIT-v4.5.4 at 95% identity and query coverage resulting in a total of 44972 contigs. A total of 44972 contigs were generated after clustering and they were further annotated with *Arabidopsis thaliana, Glycine max* and *Lycopersicum esculentum* (Supplementary Table 2). A total of 25755 contigs were annotated with *Arabidopsis thaliana, Glycine max* and *Lycopersicum esculentum* and 115, 1426 and 364 contigs were uniquely annotated (Fig. 2B). A total of 28833 contigs were annotated and 16139 contigs were unannotated.

### Functional classification and differential gene expression of *P. tetragonolobus*

In order to assign functions, contigs from HCTW and LCTW were compared against the NR protein database of *Arabidopsis thaliana*, some members of solanaceae and fabaceae family available at uniport database using blastx algorithm. To investigate the expression level of contigs in HCTW and LCTW, the numbers of clean reads were compared between libraries of each of 44972 assembled contigs through FPKM value. A total of 1235 contigs were found to be differentially expressed between the HCTW and LCTW, among them 183 contigs were up regulated and 1052 contigs were down-regulated (Supplementary Table 3).

**Figure 3:**
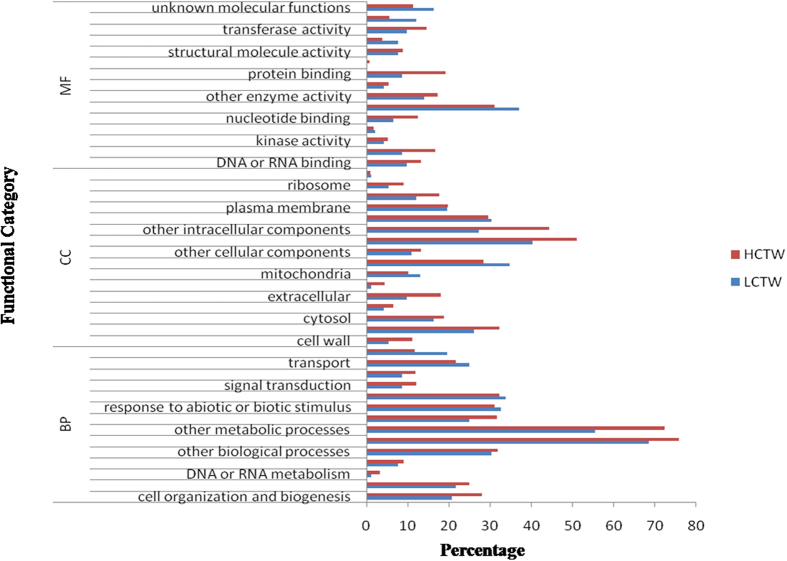
Gene ontology classification of unigenes of *P. tetragonolobus* leaf in HCTW and LCTW line.

Based on the sequence homology, 10492 sequences from HCTW and 674 sequences from LCTW were characterized into 45 functional groups under three main categories i.e. biological processes (BP), cellular component (CC) and molecular function (MF) (Fig. 3). The highest gene expression in other biological and metabolic processes in all the three GO categories followed by cellular component related genes especially ‘cell organization and biogenesis’ related gene (28.1% in HCTW and 20% in LCTW), ‘kinase activity’ is quite higher in HCTW compared to LCTW, ‘protein binding’ (19.1% in HCTW and 8.7% in LCTW), transport related pathway genes (25% in LCTW and 21% in HCTW) and response to abiotic and biotic stimulus (32.6% in LCTW and 31% HCTW) (Supplementary Table 4).

### KEGG result

To identify the biological pathways and their function in the leaf tissues of LCTW and HCTW, 44968 contigs were assembled from the contrasting lines of *P. tetragonolobus* and were mapped to the reference canonical pathways in KEGG. A total of 5210 contigs from both HCTW and LCTW lines were assigned to 229 KEGG pathways (Supplementary Table 5). 1861 contigs (35.7%) were involved in metabolic pathways, similarly, biosynthesis of secondary metabolites 995 contigs (19%), 271 contigs (5%) of plant hormone and signal transduction and 360 contigs (6.9%) of ribosome-related proteins and 154 contigs for phenylpropanoid pathway (Supplementary Table 6). As per the KEGG analysis mentioned in Supplementary Fig. 1 and Supplementary Fig. 2, the up regulated enzymes in the HCTW lines were chalcone synthase (EC2.3.1.74) and chalcone isomerase (EC5.5.1.6), peroxidases(EC1.11.1.7), cinnamyl-alcohol dehydrogeanse (EC1.1.1.195), coniferyl aldehyde dehydrogenase (EC1.2.1.68), Beta-glucosidase (EC3.2.1.21), phenylalanine ammonia lyase (EC4.3.1.24). All these enzymes and their corresponding genes were up-regulated in HCTW lines of *P. tetragonolobus*. Contrarily, S-adenosyl L-methionine: caffeic acid O-methyltransferase (EC2.1.1.68) is down-regulated in LCTW line of *P. tetragonolobus*.

**Figure 4:**
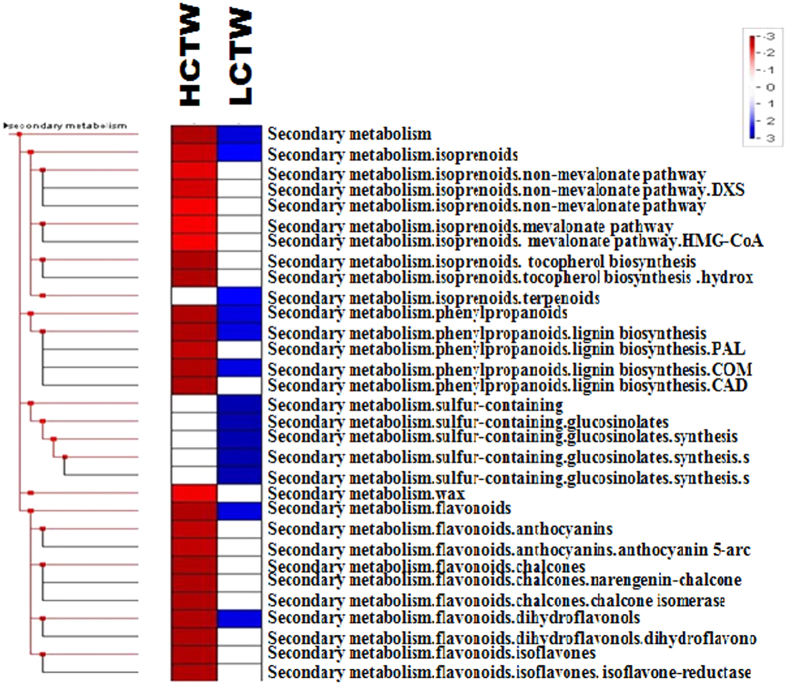
Phenyl propanoid pathway gene up and down regulated through PageMan.

PageMan analysis^21^ also showed the different expression profile of anthocyanidin-biosynthesis related pathway genes in HCTW and LCTW lines of *P. tetragonolobus* (Fig. 4). The Pageman analysis categorized the genes as: up- or down-regulated in HCTW and LCTW lines. LCTW genotype of *P. tetragonolobus* exhibited repressed or regulated flavonoid biosynthesis. Anthocyanidin biosynthesis genes were up regulated in HCTW line whereas, in LCTW line certain genes were down regulated e.g. the dihydroflavonol-4-reductase (*DFR*) gene was down regulated in LCTW line of winged bean. It had also been observed that, some genes related to unknown secondary metabolite biosynthesis like: sulphur containing glucosinolate genes were down regulated in LCTW line as compared to HCTW line. Similarly, certain isoprenoids non-mavalonate pathway related genes were up regulated in HCTW as compared to LCTW line. From these observations, it can be inferred that, there is a cross-talk of different pathways in the biosynthesis of condensed tannin. But, the present study relates its study to phenylpropanoid pathway and the expression of the genes corresponding to this pathway.

### Detection of transcription factors in winged bean

**Figure 5:**
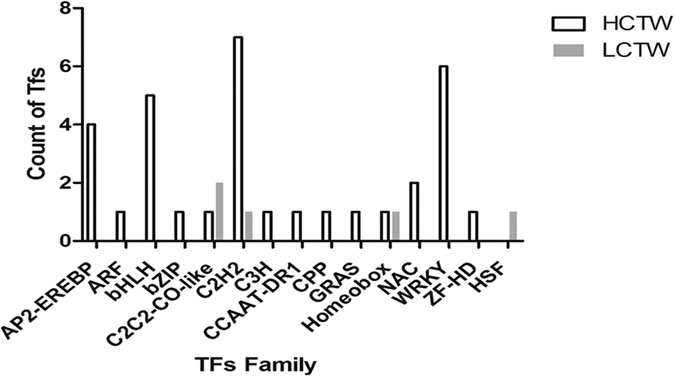
Number of Transcription factor (TF) families showed in HCTW and LCTW line of *P. tetragonolobus.*

Among all the *de novo* assembled contigs fifteen different types of transcription factor (TF) families were reported. Among these reported TFs, 33 contigs encode for HCTW and 5 contigs encode for LCTW line *P. tetragonolobus*. The maximum number of contigs were reported for C2H2 family and WRKY family TFs. The transcription factors were more frequently present in HCTW as compared to LCTW line (Fig. 5). However, bHLH group of transcription factors were only present in HCTW line of *P. tetragonolobus*.

### Identification of simple sequence repeats (SSRs)

*P. tetragonolobus* contigs of HCTW and LCTW were analyzed and 2237 and 1618 SSRs were reported between LCTW and HCTW lines respectively. Trinucleotide repeats were maximum at 881 and 663 SSRs markers in HCTW and LCTW respectively. Whereas, pentanucleotide repeats were minimum i.e. 16 and 11 in HCTW and LCTW lines respectively (Table 2).

### Genes reported for condensed tannin biosynthesis in *P. tetragonolobus*

**Figure 6:**
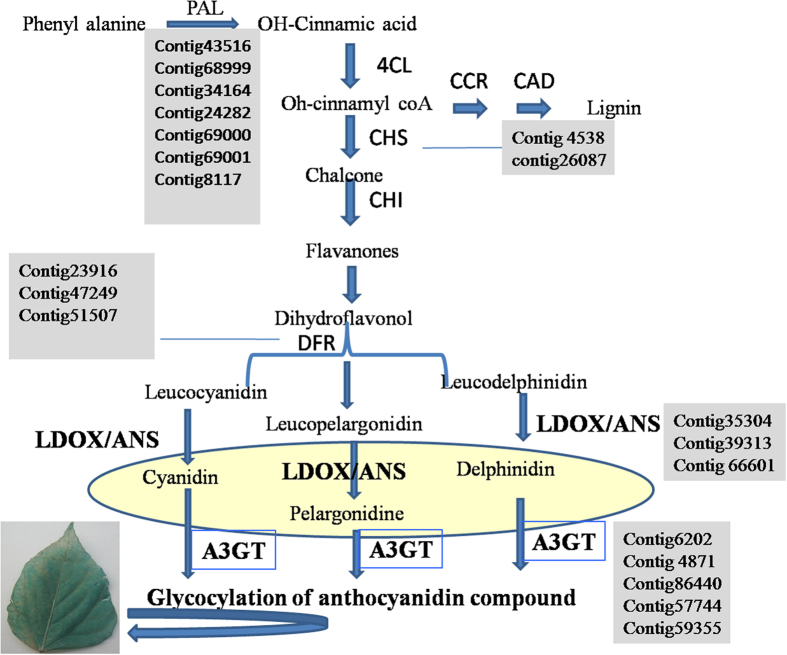
Contigs involved in flavonoid biosynthetic pathway in leaf tissue of *P. tetragonolobus.*

**Figure 7:**
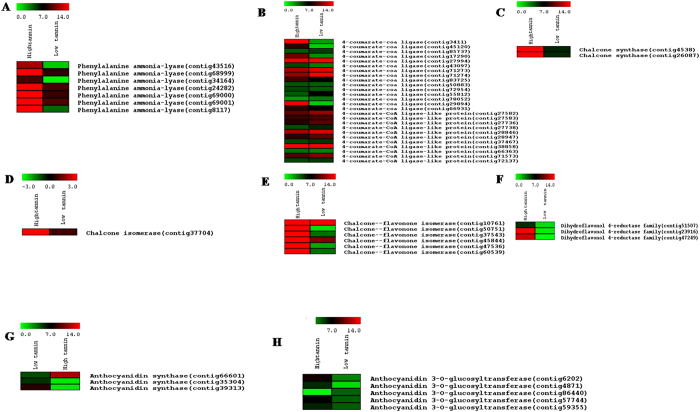
Heat map showing expression of different contigs evolved in condensed tannin biosynthesis(**A**) PAL, (**B**) 4CAL, (**C**) CHS, (**D**) CHI, (**E**) CHFI, (**F**) DFR, (**G**) ANS and (**H**) A3GT:The values in red and green indicate log2 fold increase and decrease respectively.

Precursor molecules for condensed tannin biosynthesis were reported to be derived from the phenylpropanoid pathway. Uniport annotations against members of solanaceae family were used to identify genes encoding enzymes involved in different steps of phenylpropanoid and flavonoid backbone biosynthesis in *P. tetragonolobus*. KEGG analysis showed that 10 CT-biosynthesis genes especially, *anthocyanidin synthase (ANS*), *4-coumarate-CoA ligase (4-CCL*), *chalcone synthase (CHS*), *chalcone—flavonone isomerase (CHFI*), *chalcone isomerase (CHI*), *cinnamyl alcohol dehydrogenase (CAD*), *dihydroflavonol 4-reductase (DFR*), *cinnamoyl CoA reductase (CCR*), *phenylalanine ammonia-lyase (PAL*) and *anthocyanidin 3-O-glucosyltransferase (A3GT*) had lower expression in case of LCTW in leaf tissues as compared to HCTW gene expression of *P. tetragonolobus. CHS* and *ANS* genes were more expressed in HCTW plant. A3GT gene which is involved in glycosylation of anthocyanidin compounds are responsible for biosynthesis of cyanidin compounds in plant^22^, this was also reported to have higher expression in HCTW plant. The gene analyses showed that, HCTW line have higher level of polymerization properties of condensed tannin. Among the, 26 contigs of *CCL* in which 14 contigs were highly expressed and 2 contigs were in lower expression in HCTW and 10 contigs were highly expressed and 8 contigs in lower expression in LCTW line, 3 contigs of *ANS* and 5 contigs of *A3GT* genes were highly-expressed in HCTW line as compared to LCTW and showed that the genes responsible for condensed tannin biosynthesis were highly expressed. *A3GT* and *ANS* were the major genes for cyanide compounds and its glycosylation^23^ (Fig. 6). 2, 6 and 1 contigs were present in *CHS, CHFI* and *CHI* gene respectively which is the initial enzyme for synthesis of condensed tannin and its higher expression were reported in HCTW line. *DFR* gene had 3 contigs and highly expressed in HCTW as like *ANS* contigs as *DFR* is the major responsible gene for *ANS* biosynthesis (Fig. 7). Similarly in case of *PAL* and *CAD* gene, there were 7 and 9 contigs respectively. The expression of all contigs in PAL gene were highly expressed in HCTW whereas, *CAD* contigs showed mixed type of expression but 6 contigs were highly expressed in HCTW line.

### Real time validation of the genes involved in the condensed tannin biosynthesis in *P. tetragonolobus*

**Figure 8:**
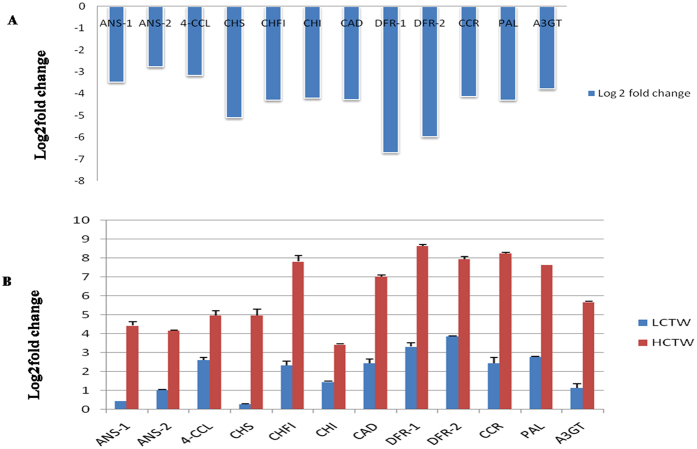
(**A**) validation of FPKM analysis of the 10 contigs involved condensed tannin biosynthesis in LCTW compared to HCTW. (**B**) Showing that qPCR validation of both HCTW and LCTW with 10 contigs.

For conformation and validation of differential expression of contrasting lines of *P. tetragonolobus* from FPKM analysis 12 contigs from phenylpropanoid pathway were chosen for qRT-PCR. These contigs were: contig_86640 (*A-3GOT*), contig_39313 (*ANS*), contig_37704 (*CHI*), contig_4538 (*CHS*), contig_50751 (*CFI*), contig_29334 (*CCR*), contig_11480 (*CAD*), contig_29894 (4*CCL*), contig_23916 (*DFR*), contig_34164 (*PAL*) which was more FPKM value in HCTW in comparative expression with LCTW contigs. In the HCTW line, mostly the genes of flavonoid biosynthesis were down regulated. The qRT-PCR data revealed the expression profile of these contigs by FPKM value (Fig. 8).

## Discussion

In this study, we explored the genes and TFs responsible for condensed tannin biosynthesis in the underutilized *P. tetragonolobus*. Each contrasting-line expressed and accumulated varying concentration of condensed tannin. Tannins are defined as a group of phenolic metabolites of varying molecular weight (500–30000 Da). Proanthocyanidins are major group of tannin which are deposited in seed and leaves of plants. Presence of high content of tannins in the presently studied wild legume seeds might be due to the metabolism of monomeric polyphenolic compounds (catechin) or polymerization of existing phenolic compounds during development and maturation^24^.

According to Serrano^25^ the estimated amount of condensed tannin for daily intake for a person is 53.6 mg/person/day. There are a lot of epidemiological data suggesting, tannin intakes which may prevent the onset of many chronic diseases. But, in case of winged bean HCTW plant seeds contained 3.5 mg/g by dry weight. If people consume it as a source of protein it will cause indigestion. Optimum intake of condensed tannins or proanthocyanidins (PAs) are considered as functional ingredients in nutritional supplements and therefore, they are presently attracting more attention.

Genome sequences of legume species of *L. japonicus*^26^, soybean^27^ and pigeonpea^28^ are now available and the corresponding data may be exploited for interpretation of transcriptome resources from unknown or less revealed data such as winged bean, to support gene annotation and comparative genome analysis. RNA-Seq technique has a lots of application for analyzing a transcript, including exploration of different biological processes of different phenotype of plant, RNA-seq technology has previously been used to characterize the transcriptomes of a number of plant species, including pea and soybean^29^,^30^. Our experiment was designed in a manner to find the genes responsible for condensed tannin biosynthesis in leaf through Illumina sequencing. Illumina sequencing (*de novo* sequencing) platform has been mostly used for transcriptome analysis of plants which has no previous reference genome^31^,^32^,^33^. Result of winged bean data and the lengths of contigs generated using Illumina platform is similar to *Vigna radiata* and *Pisum sativum* transcriptomes. They have been reported with varied lengths of 874 bp and 809 bp respectively^34^,^35^ and were recently published. The winged bean genotypes CPP34 and CPP37 also showed the average contig size is 837 and 823 bp respectively^20^.

**Figure 9:**
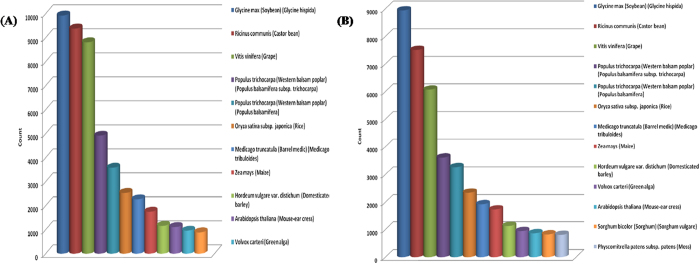
Organism sequence similarity in case of both transcript data (**A**) HCTW and (**B**) LCTW lines of *P.tetragonolobus*.

TRINITY assembler generated high number of primary assembled contigs from recently duplicated genes which lead to the generation of similar contigs^36^. The small sized sequences (~100–200 bp) may be tough to BLASTX analysis, and so the short sequences remained as unpredicted whose function was unknown^37^. Sequence similarity of contigs to the genomes and transcriptomes of other legume and tomato plant were determined using BLASTn analysis, exploring the levels of conservation up to 63%. Comparable results were observed for the winged bean transcriptome in similarity searches against other legumes^38^. Moreover, up to 63% of the annotated contigs were similar to legume genomes. BLAST analysis showed a highest level of similarity to sequences of *Glycine max*, followed by *Ricinus communis, Vitis vinifera* and *Populous trichocarpa*. The HCTW and LCTW lines also showed similarity with *Sorghum bicolor* (Fig. 9). Winged bean, *Vigna radiata* and soybean all belongs to the tropical warm-season legume, and may be mutually more closely related, within the Papilionoideae^39^.

Gene ontology (GO) is a bioinformatics-based gene functional classification system offering an updated and a strictly defined concept to comprehensively describe the properties of genes and gene products of an organism^40^. Our transcriptome data showed similar result with winged bean recent transcriptome data i.e. molecular function (MF), biological process (BP) and cellular component (CC) categories which was 46%, 37% and 16.8% respectively^20^. Kinase activity and DNA binding had more number of contigs in winged bean transcriptome showing the similar result of HCTW line of winged bean.

In case of KEGG analysis, well-represented pathways in the winged bean transcriptome included those involved in metabolic pathway, biosynthesis of secondary metabolites, flavonoid biosynthesis, carbohydrate metabolism and energy metabolism. All the major genes involved in the flavonoid biosynthesis pathway were identified and majority of them were present in HCTW lines of winged bean as compared to LCTW plant. *Vigna radiata* and *Pisum sativum* transcriptome data showed similar KEGG profile and had similar result with our data^34^,^35^. Transcription factor families were varied in both type of plant-lines. Transcription factor bHLH was predominantly present in HCTW line. Other studies showed that this TF family is most regulating factor for flavonoid biosynthesis^41^,^42^. Recently published winged bean transcriptome data of African variety showed ten transcription factors family in which WRKY family was near to similar in HCTW plant^20^. Simple sequence repeats (SSRs) marker was frequently used for diverse genetic analysis by plant breeders due to co-dominant and highly polymorphic nature^43^. Our result showed 2237 SSRs in HCTW and 1618 SSRs in LCTW, which was near to transcript data showed by other legumes^19^. Trinucleotide repeats were more predominantly present in our data like other legumes *medicago trancatula* and *Arachis hypogaea*^44^,^45^.

Accumulation of proanthocyanidin polymers within plant vacuoles is synthesized in the chloroplast and then transported to vacuoles of cell^46^. Tian (2008) reported the chalcone synthase gene expression and localization in chloroplast^47^ and Wang (2010) also localized the anthocyanidin synthase in plastid and vacuoles^48^. The gold immunolabelling experiment confirmed the presence of three enzymes of phenylpropanoid and flavonoid pathways like: Cinnamate -4-hydroxylase (C4H)^49^, chalcone synthase (CHS)^47^ and anthocyanidin reductase^48^ in the chloroplast of developing grape berries. Saslowsky and Winkel-Shirley (2001) suggested that, flavonoids are synthesized from phenylpropanoids by a multienzyme complex which is closely bound to the cytosolic face of the endoplasmic reticulum^50^. Few ultrastructural data also supports the production of phenolic compounds by the chloroplast^46^. The flavonoid monomers generally accumulated in the vacuolar storage pool^51^ through different possible routes^52^. However, the mechanism of polymerization of condensed tannins is yet to be elucidated^53^ while the nature of precursor molecules remains hypothetical^54^.

**Figure 10:**
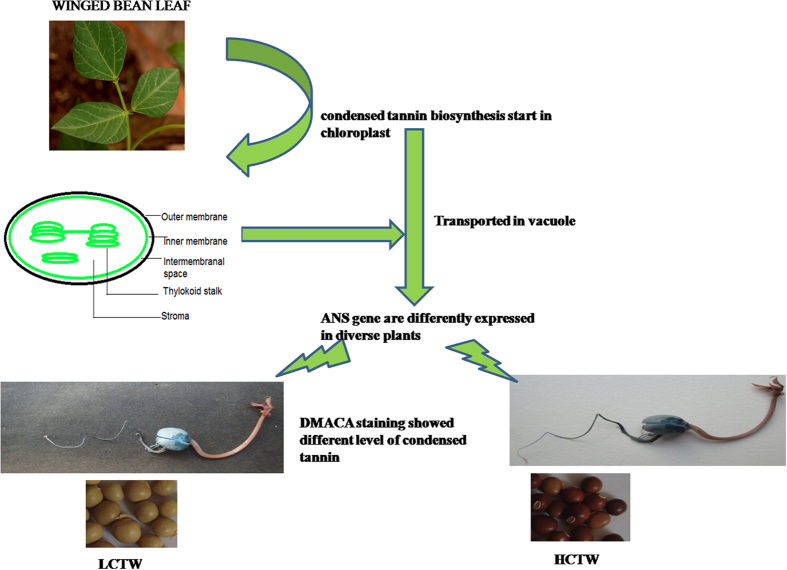
Expression of representative ANS gene showing different level of condensed tannin in different tissue. Schematic of possible mechanisms of increased level of *ANS* gene expression showed the condensed tannin deposition in HCTW line.

Some reports coined that chalcone synthase and anthocyanidin synthase are the possible precursor of condensed tannin^52^,^53^. So, we selected leaf tissue of HCTW and LCTW lines of winged bean. The seed of the contrasting lines showed variable metabolite concentration. As we have validated certain genes from leaf-tissues of *P. tetragonolobus* so, it is proposed that, the condensed tannin biosynthesis takes place in leaf tissues and subsequently might be transported to seed (Fig. 10)^46^. As transcriptome PageMan and pathway analysis showed that the anthocyanidin synthase and chalcone synthase genes were more expressed in HCTW line. Similarly, these genes were less expressed in LCTW lines of *P. tetragonolobus*.

The genes and transcription factors as well as markers identified in HCTW and LCTW lines of winged bean transcriptome study may further be utilized in generating silencing line and may be utilized for MAS breeding programme for reducing the anti-nutrient condensed tannin and improvement of this underutilized legume for enhanced consumption and utilization.

## Methods and Materials

### Plant material

Plants of *P. tetragonolobus* were procured from ICAR-National Bureau of Plant Genetic Resources, Akola, Maharashtra, India and was maintained at the botanic garden of CSIR-National Botanical Research Institute, India for further screening and experiment.

### Quantitative and qualitative estimation of condensed tannin in the plant

#### Vanillin assay

The total condensed tannin was determined by colorimetric method as well as modified vanillin-hydrochloric acid assay developed by Hagerman^55^. The deduced value was confirmed thrice. The highest and lowest condensed tannin-containing lines of *P. tetragonolobus* were selected for further study and anlaysis.

#### HPLC analysis of leaf extracts

The leaves of high and low-CT containing-lines (HCTW i.e. High condensed tannin winged bean and LCTW i.e low condensed tannin winged bean) were quantified by standard HPLC method^56^. The standards cyanidin, pelargonidin and delphinidin were used for further compositional quantification.

#### Histochemical staining with dimethylamminocinnamaldehyde (DMACA)

The histochemical staining of condensed tannin (CT) in leaves was carried out by staining tissues in a solution of ethanol: 6 M HCl (1:1) containing 0.1% (w/v) dimethylamminocinnamaldehyde (DMACA) (Sigma-Aldrich) for 3 to 6 min^57^.

#### RNA isolation, library preparation and sequencing

Leaf tissues of diverse CT- containing lines (VS1, VS2 biological replicate of HCTW and VS3, VS4 biological replicates of LCTW) of *P. tetragonolobus* were collected from two months old plants grown in the garden of CSIR-National Botanical Research Institute. RNA was isolated from leaf tissues through RNA isolation kit (Sigma). The isolated RNA was treated with DNase enzyme for removal of contaminant DNA. The quality and quantity of total RNA was calculated through Bioanalyzer (Agilent). Good quality RNA (RIN value > 7) was further subjected to analysis and preparation of cDNA. The cDNA were amplified according to Illumina True-Seq protocol and sequenced on Illumina Nextseq500 platform. The samples were run in duplicates. The transcriptome library for sequencing was carried out according to Illumina Trueseq RNA sample preparation guide (part # 1004898). The enriched poly A RNA (1000 ng) was prepared using RNA purification beads which were fragmented for 4 min at elevated temperature (94 °C) in presence of divalent cations and reverse transcribed with superscript III reverse transcriptase by priming with random hexamers. Second strand cDNA was synthesized in presence of DNA polymerase I and RNaseH. The cDNA was cleaned up using Agencourt Ampure XP SPRI beads (Beckman Coulter, USA) followed by ligation of “Illumina adapters” to the cDNA molecules after end repair and addition of “A”-base. Following SPRI cleanup after ligation, the library was amplified using 11 cycles of PCR for enrichment of adapter ligated fragments. This method of library-preparation had been followed as per the manufacturer’s protocol. The prepared library was further quantified using nanodrop and validated for quality by running an aliquot on high sensitivity bioanalyzer chip (Agilent). There were total four libraries prepared for further processing and analysis. Two libraries for high condensed tannin-containing winged bean HCTW (VS1, VS2) and the other two libraries for low condensed tannin-containing winged bean LCTW (VS3, VS4).

#### *De novo* assembly and annotation

Raw reads which were generated after sequencing were in fastq file format. The fastq files were trimmed to remove adapter sequences using fastq-mcf tool. The fastq file were filtered to remove reads having average quality score less than 20 in any of the paired end data. The trimmed reads were then *de novo* assembled using TRINITY^58^ with default options. The raw reads data is available at the NCBI public domain with accession number SRA293442 for HCTW variety (VS1 and VS2) and SRA293454 for LCTW variety (VS3-VS4). The assembled contigs were annotated with the NCBI-NR and TAIR10 protein database using BLASTX with e-value < 10^−5^. The data was also annotated with *Lycopersicum esculentum* and *Glycine max* genomic database with same parameters. Non-redundant sequence dataset was generated using CD-HIT (Cluster Database at High Identity with Tolerance) by removing homologous sequences with identity threshold of (0.95 or 95%).

#### Sequence annotation and functional characterization

Differential gene expression analysis was performed using DEGseq program and FDR value was retrieved from q-value program in R-Bioc. The contigs with *p*-value < 0.05, FDR < 0.05 and Fold change <−1 or >1 were selected. The GO annotation was performed using the TAIR10 IDs for the annotated contigs using agriGO (http://bioinfo.cau.edu.cn/agriGO/analysis.php). The GO annotation was retrieved using singular enrichment analysis (SEA) statistical test at *p*-value < 0.05.

#### Biological pathway annotation

The PageMan analysis was performed using the differential genes which were annotated with the TAIR10 protein database. The analysis was performed with Benjamini Hochberg multiple testing correction and average statistics method. All the TAIR10 IDs of single assemblies were used to extract the KO IDs from the KAAS database (http://www.genome.jp/tools/kaas/). These KO IDs were submitted in KEGG (http://www.genome.jp/kegg/) to extract all the metabolic pathways^59^.

#### Detection of SSR molecular markers

Total Contig of HCTW and LCTW were analyzed with using Micro-satellite identification tool (MISA) for identification of simple sequence repeats (SSRs) having mononucleotides to hexanucleotides repeats.

#### Validation of gene expression by RT-PCR

Total RNA from both plants was extracted and treated with RNase-free DNase I (Ambion). First-strand complementary DNA was synthesized using 5 μg of total RNA with oligo (dT) primer (Fermentas). The qRT-PCR was done in the Step OnePlus Real-Time PCR System (Applied Biosystems 7500). Primers used for qRT-PCR are listed in Supplementary Table 7. Contig abundances were calculated using Cufflinks^60^ ver.0.93 and the output normalized expression values in FPKM (Fragments per kilo-base of exon per million fragments mapped) analogous to single-read FPKM^61^ were used for further comparative analysis. To confirm the expression level using FPKM, 12 genes were chosen as cases for PCR validation. Each reaction contained cDNA (1 μl) and Fast SYBR Green master Mix (Applied Biosystem, USA) 10 μl in a final volume of 20 μl. The reactions were performed using the following cycle conditions, an initial 94 °C for 2 min, followed by 30 cycles of 94 °C for 30 s, 60 °C for 30 s, and 72 °C for 30 s, and the final 5 min extension at 72 °C. The mRNA levels were normalized with 18 s rRNA gene^62^. All experiments were used in triplicates for each sample and relative gene expression levels were calculated using 2^−ΔΔCT^ method.

## Additional Information

**Accession codes:** Transcriptome data of both diverse genotype of winged bean supporting the conclusion of the article are available in NCBI SRA repository as an accession no. SRA293442 for HCTW and SRA293454 for LCTW plant. Dataset of these transcriptome are available as excel file.

**How to cite this article:** Singh, V. *et al. De novo* sequencing and comparative analysis of leaf transcriptomes of diverse condensed tannin-containing lines of underutilized *Psophocarpus tetragonolobus* (L.) DC. *Sci. Rep.* **7**, 44733; doi: 10.1038/srep44733 (2017).

**Publisher's note:** Springer Nature remains neutral with regard to jurisdictional claims in published maps and institutional affiliations.

## Supplementary Material

Supplementary Information

Supplementary Table 2

Supplementary Table 3

Supplementary Table 4

Supplementary Table 5

Supplementary Table 6

## Figures and Tables

**Table 1:** Summary of annotation in HCTW (high condensed tannin winged bean) and LCTW (low condensed tannin winged bean) of *P. tetragonolobus.*

	HCTW		LCTW	
	VS1	VS2	VS3	VS4
Total No. of Reads	13901780	30155788	9090662	10394022
Total No. of HQ Reads	12604265 (84.01%)	27769919 (92.09%)	8098380 (89.08%)	9518980 (91.58%)
	**VS1_VS2**		**VS3_VS4**	
Total contigs	102586		88433	
Range of assembled contigs (bp)	151–87925		151–69464	
Average contig length (bp)	713.192		769.002	
N50	1466		1567	
GC%	57.15		58.92	
	**Combined assembly**			
Total contigs	44968			
Range of assembled contigs (bp)	151–87925			
Average contig length (bp)	1247.077			
GC%	39.33			
TAIR10 Annotation contigs	26438			
TAIR10 Unannotated contigs	18530			
Gmax Annotation contigs	28171			
Gmax Unannotated contigs	16797			
tomato Annotation contigs	26907			
tomato Unannotated contigs	18061			

**Table 2:** SSRs detected of LCTW and HCTW through MISA.

Type of winged bean	HCTW(VS1_VS2)	LCTW(VS3_VS4)
Total number of sequences examined	69338	59253
Total number of identified SSRs	2237	1618
p1 (Mono nucleotide Repeats)	622	453
p2 (Di nucleotide Repeats)	532	355
p3 (Tri nucleotide Repeats)	881	663
p4 (Tetra nucleotide Repeats)	23	17
p5 (Penta nucleotide Repeats)	16	11
p6 (Hexa nucleotide Repeats)	59	50
Number of compound SSR (i e c)	102	67
c* (Type of compound SSR)	2	1

## References

[b1] Amoo I. A., Adebayo O. T. & Oyeleye A. O. Chemical evaluation of winged beans (*Psophocarpus tetragonolobus*), Pitanga cherries (*Eugenia uniflora*) and orchid fruit (Orchid fruit *myristica*). Afr. J. Food Agric. Nutr. Dev. 6, 1–12 (2006).

[b2] Anon. The winged bean, A high-protein crop for the tropics. (2nd Edition) National Academy Press, Washington DC, 46. (1981).

[b3] Khan T. N. Winged bean production in the tropics. FAO Plant Production and Protection Paper 38, 222 (1982).

[b4] National Research Council (US). Underexploited Tropical Plants with Promising Economic Value. 2nd Edition. US National Academies (1975).

[b5] De Lumen B. O. & Salamat L. A. Trypsin inhibitor activity in winged bean (Psophocarpus tetragonolobus) and the possible role of tannin. J Agric Food Chem. 28, 533–536(1980). 7391398 10.1021/jf60229a042

[b6] Tan N. H., Rahim Z. H. A., Khor H. T. & Wong K. C. Winged bean (*Psophocarpus tetragonolobus*) tannin level, phytate content and hemagglutinating activity. J. Agric. Food Chem. 31, 916–917 (1983). 6619430 10.1021/jf00118a063

[b7] Kantha S. S., Hettiarachchy H. S. & Erdman J. W. Nutrient, anti-nutrient contents and solubility profiles of nitrogen, phytic acid and selected minerals in winged bean flour. Cereal Chemistry 63, 9–13 (1986).

[b8] Klu G., Jacobsen E. & Vanttarten A. M. Induced mutation in winged bean psophocarpous tetragonolobus L.DC.) with low tannin content. Euphytica. 98, 99–107 (1997).

[b9] Heller W. & Forkmann G. Biosynthesis. In: J. B. Harborne, The Flavonoids. pp. 399–425. Chapman & Hall, London (1988).

[b10] Van der Meer I. Regulation of flavonoid gene expression in *Petunia hybrida*: CIS-Acting elements and Trans-acting factors. Ph.D Thesis. pp 146. Vrije Universiteit, Amsterdam (1991).

[b11] Martin C. & Gerats T. The control of pigment biosynthesis during petal development. Plant Cells 5, 1253–1264 (1993). 10.1105/tpc.5.10.1253 PMC160358 12271025

[b12] Mol J. N. M. Molecular biology of anthocyanin biosynthesis. In: Polyphenolic Phenomena. A. Scalbert (Paris, INRA) pp 87–98 Washington (1993)

[b13] Koes R. E., Quattrocchio F. & Mol J. N. M. The flavonoid biosynthetic pathway in plants: Functions and evolution. BioEssay 16, 123–132 (1994).

[b14] Koes R., Verweij W. & Quattrocchio F. Flavonoids: a colorful model for the regulation and evolution of biochemical pathways. Trends Plant Sci. 10, 236–242 (2005). 15882656 10.1016/j.tplants.2005.03.002

[b15] Bais H. P., Vepachedu R., Gilroy S., Callaway R. M. & Vivanco J. M. Allelopathy and exotic plant invasion: from molecules and genes to species interactions. Science. 301, 1377–1380 (2003). 12958360 10.1126/science.1083245

[b16] Santos-Buelga C. & Scalbert A. Proanthocyanidins and tannin-like compounds – nature, occurrence, dietary intake and effects on nutrition and health. J. Sci. Food Agric. 80, 1094–1117 (2000).

[b17] Pritchard D. A. . The effect of polyethylene glycol (PEG) on wool growth and live weight of sheep consuming a mulga (*Acacia aneura*) diet. Proceedings of the Australian Society of Animal Production. 17, 290–294 (1988).

[b18] Rastogi S. . De novo sequencing and comparative analysis of holy and sweet basil transcriptomes. BMC genomics. 15, 588, doi: 10.1186/1471-2164-15-588 (2014). 25015319 PMC4125705

[b19] Chapman M. A. Transcriptome sequencing and marker development for four underutilized legumes. Appl. Plant Sci. 3, apps. 1400111, doi: 10.3732/apps.1400111 (2015). PMC4332146 25699221

[b20] Vatanparast M. . Transcriptome sequencing and marker development in winged bean (*Psophocarpus tetragonolobus*;leguminosae). Scientific reports. 6, 29070, doi: 10.1038/srep29070 (2016). 27356763 PMC4928180

[b21] Goffard N. & Weiller G. Functional analysis of legume genome arrays. Methods in Molecular Biology. 1069, 59–66 (2013). 23996308 10.1007/978-1-62703-613-9_5

[b22] Kamsteeg J., van Brederode J. & van Nigtevecht G. Identification and properties of UDP-glucose: cyanidin-3-O-glucosyltransferase isolated from petals of the red campion (*Silene dioica*). Biochem. Genet. 16 (11–12), 1045–58 (1978). 751640 10.1007/BF00484525

[b23] Dixon R. A., Xie D. Y. & Sharma S. B. Proanthocyanidins – a final frontier in flavonoid research? New Phytologist. 165, 9–28, doi: 10.1111/j.1469-8137.2004.01217.x (2005). 15720617

[b24] Chavan U. D., Shahidi F. & Naczk M. Extraction of condensed tannins from beach pea (Lathyrus maritimus L.) as affected by different solvents. Food Chemistry. 75, 509–512 (2001).

[b25] Serrano J., Pimi R. P., Dauer A., Aura A. M. & Calixto F. X. Tannins: Current knowledge of food sources, intake, bioavailability and biological effects. Mol. Nutr. Food Res. 53, S310–S329, doi: 10.1002/mnfr.200900039 (2009). 19437486

[b26] Sato S. . Genome structure of the legume, *Lotus japonicus*. DNA Res. 15, 227–39 (2008). 18511435 10.1093/dnares/dsn008 PMC2575887

[b27] Schmutz J. . Genome sequence of the palaeopolyploid soybean. Nature. 463, 178–83 (2012). 10.1038/nature08670 20075913

[b28] Varshney R. K.. Draft genome sequence of pigeonpea (*Cajanus cajan*), an orphan legume crop of resource-poor farmers. Nat Biotech. 30, 83–9 (2011). 10.1038/nbt.2022 22057054

[b29] Alves-Carvalho S. . Full-length de novo assembly of RNA-seq data in pea (*Pisum sativum* L.) provides a gene expression atlas and gives insights into root nodulation in this species. Plant J. 84, 1–19, doi: 10.1111/tpj.12967 (2015). 26296678

[b30] Severin A. J. . RNA-Seq atlas of *Glycine max*: a guide to the soybean transcriptome. BMC Plant Biol. 14, 160 (2015). 10.1186/1471-2229-10-160 PMC3017786 20687943

[b31] Wang Z. . De novo assembly and characterization of root transcriptome using Illumina paired-end sequencing and development of cSSR markers in sweet potato (*Ipomoea batatas*). BMC Genomics. 11, 726 (2010). 21182800 10.1186/1471-2164-11-726 PMC3016421

[b32] Annadurai R. S. . *De novo* transcriptome assembly (NGS) of *Curcuma longa* L. rhizome reveals novel contigs related to anticancer and antimalarial terpenoids. PLoS One. 8(2), e56217 (2013). 23468859 10.1371/journal.pone.0056217 PMC3585318

[b33] Kim H. A. . High-throughput sequencing and *de novo* assembly of *Brassica oleracea var. Capitata* L. for transcriptome analysis. PLoS One. 9(3), e92087 (2014). 24682075 10.1371/journal.pone.0092087 PMC3969326

[b34] Chen H. . Transcriptome sequencing of Mung Bean (*Vigna radiate* L.) Genes and the identification of EST-SSR Markers. PLosOne. 10(4), e0120273, doi: 10.1371/journal.pone.0120273 (2015). PMC4382333 25830701

[b35] Sudheesh S. . *De novo* assembly and characterisation of the field pea transcriptome using RNA-seq. BMC Genomics. 16, 611(2015). 26275991 10.1186/s12864-015-1815-7 PMC4537571

[b36] Grabherr M. G. . Full-length transcriptome assembly from RNA-seq data without a reference genome. Nat. Biotechnol. 15, 29(7), 644–52, doi: 10.1038/nbt.1883 (2011). PMC3571712 21572440

[b37] Li D., Deng Z., Qin B., Liu X. & Men Z. *De novo* assembly and characterization of bark transcriptome using Illumina sequencing and development of EST-SSR markers in rubber tree (*Hevea brasiliensis Muell. Arg*.). BMC Genomics. 13, 192 (2012). 22607098 10.1186/1471-2164-13-192 PMC3431226

[b38] Varshney R. K. . Draft genome sequence of chickpea (*Cicer arietinum*) provides a resource for trait improvement. Nat Biotech. 31, 240–6 (2013). 10.1038/nbt.2491 23354103

[b39] Doyle J. J. & Luckow M. A. The rest of the iceberg Legume diversity and evolution in a phylogenetic context. Plant Physiol. 131, 900–10, doi: 10.1104/pp.102.018150 (2003). 12644643 PMC1540290

[b40] Gahlan P. . *De novo* sequencing and characterization of *Picrorhiza kurrooa* transcriptome at two temperatures showed major transcriptome adjustments. BMC Genomics. 13, 126 (2012). 22462805 10.1186/1471-2164-13-126 PMC3378455

[b41] Hichri I. . Recent advances in the transcriptional regulation of the flavonoid biosynthetic pathway. Journal of Experimental Botany. 62, 2465–2483 (2011). 21278228 10.1093/jxb/erq442

[b42] Feller A., Machemer K., Braun E. L. & Grotewold E. Evolutionary and comparative analysis of MYB and bHLH plant transcription factors. Plant Journal. 66, 94–116 (2011). 21443626 10.1111/j.1365-313X.2010.04459.x

[b43] Wang M. L., Barkley N. A. & Jenkins T. M. Microsatellite markers in plants and insects. Part I: Applications of biotechnology. G33, 54–67 (2009).

[b44] Eujayl I. . Medicago truncatula EST-SSRs reveal cross-species genetic markers for Medicago spp. Theor. Appl. Genet. 108, 414–422, doi: 10.1007/s00122-003-1450-6 (2004). 13679975

[b45] Bosamia T. C., Mishra G. P., Thankappan R. & Dobaria J. R. Novel and stress relevant EST derived SSR markers developed and validated in peanut. PLoS One. 10, e0129127, doi: 10.1371/journal.pone.0129127 (2015). 26046991 PMC4457858

[b46] Brillouet J. M. . The tannosome is an organelle forming condensed tannins in the chlorophyllous organs of Tracheophyta. Ann Bot. 1–12, doi: 10.1093/aob/mct168 (2013). PMC3783233 24026439

[b47] Tian L., Wan S. B., Pan Q. H., Zheng Y. J. & Huang W. D. A novel plastid localization of chalcone synthase in developing grape berry. Plant Science. 175, 431–436 (2008).

[b48] Wang H. . Gene transcript accumulation, tissue and subcellular localization of *anthocyanidin synthase (ANS*) in developing grape berries. Plant Science. 179, 103–113 (2010).

[b49] Chen J. Y., Wen P. F., Kong W. F., Pan O. H., Wan S. B. & Huang W. D. Changes and subcellular localizations of the enzymes involved in phenylpropanoid metabolism during grape berry development. J Plant Physiol. 163, 115–127 (2006). 16399002 10.1016/j.jplph.2005.07.006

[b50] Saslowsky D. & Winkel-Shirley B. Localization of flavonoid enzymes in Arabidopsis roots. The Plant Journal. 27, 37–48 (2001). 11489181 10.1046/j.1365-313x.2001.01073.x

[b51] Debeaujon I., Peeters A. J., Leon-Kloosterziel K. M. & Koornneef M. The TRANSPARENT TESTA12 gene of Arabidopsis encodes a multidrug secondary transporter-like protein required for flavonoid sequestration in vacuoles of the seed coat endothelium. The Plant Cell. 13, 853–871 (2001). 11283341 10.1105/tpc.13.4.853 PMC135529

[b52] Zhao J., Pang Y. & Dixon R. A. Update on biosynthesis of proanthocyanidins. The mysteries of proanthocyanidin transport and polymerization. Plant Physiology. 153, 437–443 (2010). 20388668 10.1104/pp.110.155432 PMC2879784

[b53] Zhao J. & Dixon R. A. MATE transporters facilitate vacuolar uptake of epicatechin3′-O-glucoside for proanthocyanidin biosynthesis in *Medicago truncatula* and *Arabidopsis*. The Plant Cell. 21, 2323–2340 (2009). 19684242 10.1105/tpc.109.067819 PMC2751950

[b54] Pang Y. . Medicago glucosyltransferase UGT72L1: potential roles in proanthocyanidin biosynthesis. Planta. 238, 139–154 (2013). 23592226 10.1007/s00425-013-1879-z

[b55] Hagermann A. E. The Tannin handbook. USA: oxford OH 45056 (2002).

[b56] Niranjan A. . Development and vali-dation of HPLC-UV-MS/MS method for identification and quantification of polyphenols in *Artemisia pallens* L. Acta Chromatographica. 21, 105–116, doi: 10.1556/AChrom.21.2009.1.9 (2009).

[b57] Porter L. J. Tannins in Methods in plant Biochemistry (plant phenolics), eds Dey P. M. & Harborne J. B., Academic Press, London, 1, 389–419 (1989).

[b58] Haas B. J. . *De novo* transcript sequence reconstruction from RNA-seq using the Trinity platform for reference generation and analysis. Nat Protoc. 8, 1494–1512 (2013). 23845962 10.1038/nprot.2013.084 PMC3875132

[b59] Kanehisa M., Sato Y., Kawashima M., Furumichi M. & Tanabe M. KEGG as a reference resource for gene and protein annotation. Nucleic Acids Res. 44, D457–D462 (2016). 26476454 10.1093/nar/gkv1070 PMC4702792

[b60] Trapnell C. . Differential gene and transcript expression analysis of RNA-seq experiments with TopHat and Cufflinks, Nat Protoc. 7(3), 562–78, doi: 10.1038/nprot.2012.016 (2012). 22383036 PMC3334321

[b61] Mortazavi A., Williams B. A., McCue K., Schaeffer L. & Wold B. Mapping and quantifying mammalian transcriptomes by RNA-Seq. Nat Methods. 5(7), 621–8, doi: 10.1038/nmeth.1226 (2008). 18516045 PMC13303166

[b62] Nicot N., Hausman J. F., Hoffmann L. & Evers D. Housekeeping gene selection for real-time RT-PCR normalization in potato during biotic and abiotic stress. J Exp Bot. 56(421), 2907–2914, doi: 10.1093/jxb/eri285 (2005). 16188960

