# Supplementary material for: De novo sequencing and comparative analysis of leaf transcriptomes of diverse condensed tannin-containing lines of underutilized Psophocarpus tetragonolobus (L.) DC
Source: Sci Rep. 2017 Mar 21;7:44733. doi: 10.1038/srep44733 (PMC5359716; doi:10.1038/srep44733)

***De novo* sequencing and comparative analysis of leaf transcriptomes of diverse condensed tannin-containing lines of underutilized *Psophocarpus tetragonolobus* (L.) DC.**

Vinayak Singh[1,2], Ridhi Goel[1], Veena Pande[2], Mehar Hasan Asif[1], Chandra Sekhar Mohanty[1]*

Affiliation:

[1]Plant Molecular Biology and Genetic Engineering Division,
CSIR-National Botanical Research Institute,
Lucknow-226 001
Uttar Pradesh, India.

[2]Department of Biotechnology,
Kumaun University,Nainital,
Uttarakhand,India.

Email Address:
Vinayak Singh :visiranj@gmail.com
Ridhi Goel:ridhi.goel2003@gmail.com
Veena pande: veena_biotech@rediffmail.com
Mehar Hasan Asif: mh.asif@nbri.res.in

*Corresponding author
Chandra Sekhar Mohanty
E-mail: cs.mohanti@nbri.res.in, sekhar_cm2002@rediffmail.com
Tel. No.: (0522)22058937
FAX: (0522)22058836

Supplementary materials:

Supplementary Table 1. Geographical distribution of winged bean germplasm and vanillin assay result showed two diverse germplasm

Supplementary Table 2. Annotaion of transcript with *A.thaliana*, *G.max* and *S.lycopersicum* (Supplementary table 2..xlsx)

Supplementary Table 3.Diffentialy expressed gene of HCTW and LCTW winged bean lines (Supplementary table 3..xlsx)

Supplementary Table 4. Functional classification of transcriptome data (Supplementary table 4..xlsx)

Supplementary Table 5.KEGG pathway result (Supplementary table 5..xlsx)

Supplementary Table 6. Kobas analysis result of KEGG pathway (Supplementary table 6..xlsx)

Supplementary Table 7. Real time PCR primer for validation of transcriptome data

Supplementary Table 1

Winged bean germplasm

**Accession No.**	**Country**	**CONCENTRATION (mg/g)**
IC 26942	India	3.56
IC 26944	India	1.455
IC 26944-1	India	0.71
IC 26945	India	1.71
IC 26946	India	1.602
IC 26949	India	1.561
IC 26949-1	India	0.293
IC 31981	India	1.606
IC 34861	India	1.138
IC 41980	India	0.868
IC 95228	India	2.359
IC 95229	India	0.408
IC 95230	India	1.686
IC 95231	India	1.689
IC 95232	India	1.876
IC 95235	India	3.315
IC 95236	India	2.272
IC 95237	India	0.935

IC 95237-1	India	2.452
IC 95238	India	0.613
IC 95239	India	1.513
IC 95241	India	0.912
IC 95242	India	0.824
IC 112416	India	0.638
EC 11885		0.849
EC 21904	Ghana	2.581
EC 27885-1	Ghana	2.078
EC 27885-2	Ghana	0.814
EC 27886-A	Ghana	3.452
EC 38154		1.929
EC 38821	Papua New Guinea	3.671
EC 38821 PA	Papua New Guinea	1.008
EC 38821 P-1	Papua New Guinea	1.312
EC 38821 P2-1	Papua New Guinea	0.657
EC 38821-B	Papua New Guinea	1.42
EC 38821-1	Papua New Guinea	2.499
EC 38821-2	Papua New Guinea	1.243
EC 38824-2	Papua New Guinea	1.513
EC 38825	Papua New Guinea	2.029
EC 38954-A	Papua New Guinea	3.665
EC 38955-B	Papua New Guinea	2.385
EC 38956	Papua New Guinea	0.386
EC 38956-1(HCTW)	Papua New Guinea	3.796
EC 38956-2	Papua New Guinea	1.39
EC 38956-3	Papua New Guinea	1.373
EC 38957	Papua New Guinea	2.3
EC 38957-A	Papua New Guinea	2.518
EC 38957-B	Papua New Guinea	1.159
EC 38958	Papua New Guinea	3.566
EC 38959	Papua New Guinea	2.699
EC 130184	Indonesia	2.193
EC 130184-2	Indonesia	1.479
EC 142600	Philippines	2.709
EC 178267	Thailand	1.641
EC 178268(LCTW)	Thailand	0.265

EC 178269	Thailand	2.008
EC 178271	Thailand	3.372
EC 178272	Thailand	0.643
EC 178274	Thailand	2.546
EC 178275-1	Thailand	1.862
EC 178277	Thailand	2.316
EC 178278	Thailand	1.138
EC 178279	Thailand	1.408
EC 178282	Thailand	3.095
EC 178283	Thailand	2.438
EC 178284	Thailand	2.227
EC 178286	Thailand	0.812
EC 178287	Thailand	0.032
EC178288	Thailand	1.393
EC 178289	Thailand	0.608
EC 178289-1	Thailand	0.162
EC 178291	Thailand	1.401
EC 178292	Thailand	3.56
EC 178293	Thailand	1.604
EC 178295	Thailand	2.001
EC 178296	Thailand	0.719
EC 178297	Thailand	0.929
EC 178298	Thailand	1.006
EC 178299	Thailand	0.609
EC 178301	Thailand	1.539
EC 178302	Thailand	0.485
EC 178303	Thailand	2.005
EC 178304	Thailand	0.994
EC 178305	Thailand	1.184
EC 178306	Thailand	1.365
EC 178307	Thailand	0.694
EC 178308	Thailand	2.588
EC 178309	Thailand	2.651
EC 178310	Thailand	1.142
EC 178311	Thailand	1.036
EC 178312	Thailand	0.638
EC 178313	Thailand	1.485

EC 178314	Thailand	0.393
EC 178318	Thailand	1.144
EC 178319	Thailand	2.037
EC 178322	Thailand	0.864
EC 178325	Thailand	0.295
EC 178332	Thailand	1.388
EC 178335	Thailand	2.25
AKWB-1	Selection from Indonesian germplasm EC 114273-B	3.681

Supplementary Table 7. Real time PCR primer for validation of transcriptome data

**S.No.**	**Gene**	**Forward Primer**	**Reverse primer**
1.	ANS1	AAGCACTAACTGAATCACCTGAACTC	CCACTATTTCATCATCTGGATTGG
2.	ANS2	TGGGTTCAGGGAAGCATCAG	ATCCAGAATCTAGGTTCATTGCATT
3.	CCL	TCGATGAGGGCGGGTTT	CCTCGTTTCCCACCTCTTCA
4.	CHS	CACCTGACGGAGGAGTTTCTG	CGACGGCGCCATGTAAG
5.	CHF	TGGAGGAACCAGCAACAAATG	TTGAGCAACCCGGGAAAC
6.	CHI	TCCGACCGCCCCTTTC	AGCCCAGAGAGGCAACGA
7.	CAD	TCCCTGTGAAGGCTTTTGGT	CAACAATTTCATGCCTTCTGGAA
8.	DFR1	GAAGCATCTGGTGTTCGTTTTTG	TCTTGAAAGGCTATATGAACTTGTTTCT
9.	DFR2	GTGGTGGATGAGAGTGTTTGGA	TGACCAACTAAAGGGCTTCACA
10.	CCR	CAAAGATGGCAAAAGATTCACAAA	TCTCTAGCAGCCCAACCAAAA
11.	PAL	GCTGCCGCTATTATGGAACAC	GGCTGTTGCAGTTTAGCATTTTT
12.	A3GT	GATTCGCCGCTCGCCTAC	GGTTTCCCATATTGTTTTGCGA
13.	18SrRNA	ATTCTATGGGTGGTGGTGC	CCATCCAATCGGTAGGAGC

Supplementary figures.

Figure 1: **KEGG analysis of phenylpropanoid pathway**: highly expressed genes in HCTW line and low expressed genes in LCTW line.

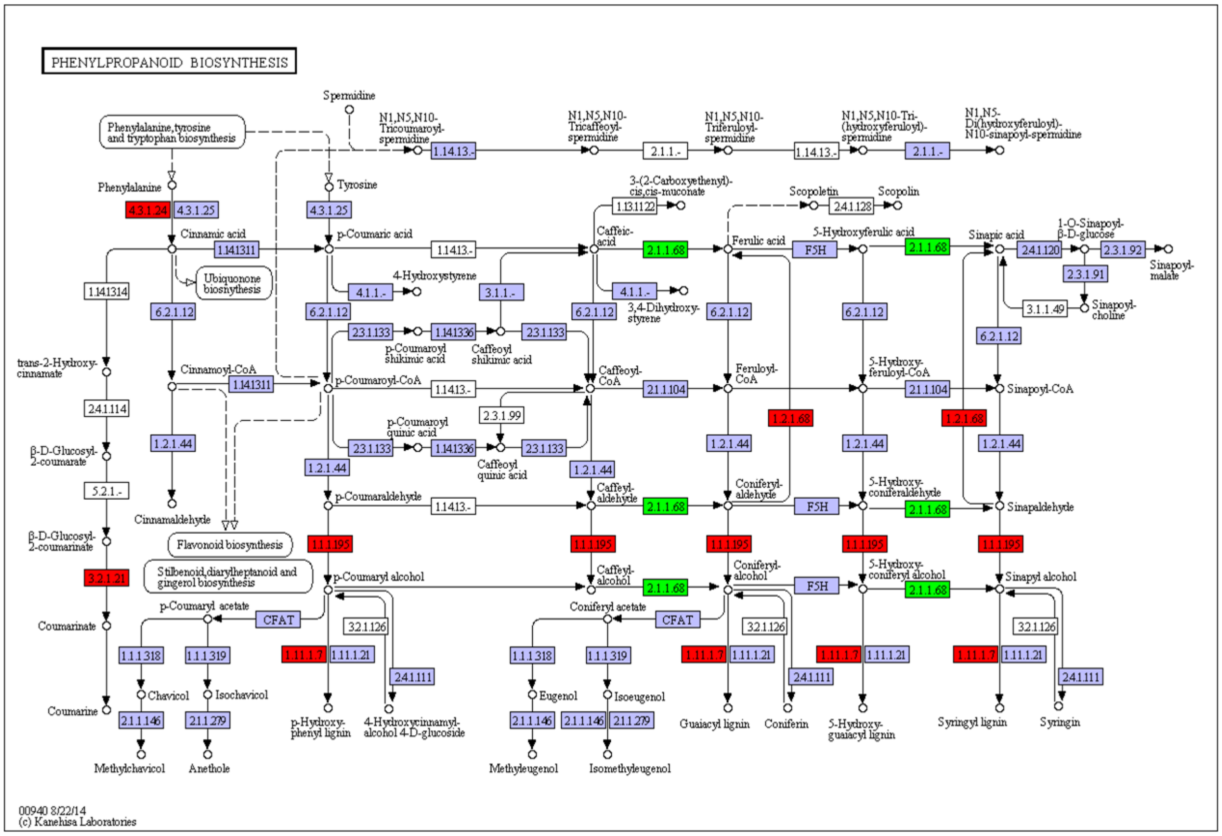

Figure 2: KEGG analysis of flavonoid biosynthesis: flavonoid biosynthesis genes found in HCTW line of *P.tetragonolobus* showed higher expression of gene

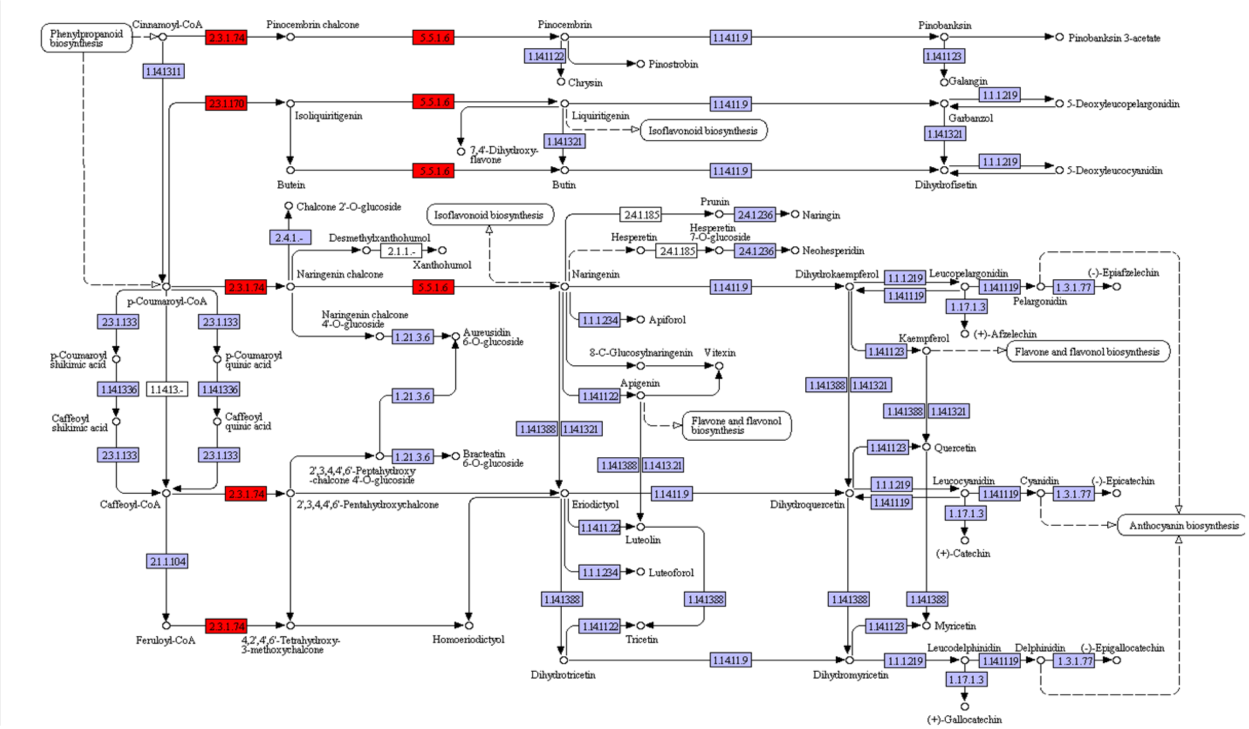

Supplement: Supplementary Information [file srep44733-s1.pdf]
